# Gallic acid and exercise training improve motor function, nerve conduction velocity but not pain sense reflex after experimental sciatic nerve crush in male rats

**Published:** 2015

**Authors:** Maryam Hajimoradi, Mohammad Fazilati, Mohammad Kazem Gharib-Naseri, Alireza Sarkaki

**Affiliations:** 1 *Department of Biology, Faculty of Basic Sciences, Isfahan Payamenoor University, Isfahan, Iran.*; 2 *Department of Biochemistry, Faculty of Basic Sciences, Payamnoor University, Tehran, Iran*; 3 *Ahvaz Physiology Research Center, Ahvaz Jundishapur University of Medical Sciences, Ahvaz, Iran.*; 4 *Department of Physiology, School of Medicine, Ahvaz Jundishapur University of medical Sciences, Ahavaz, Iran*

**Keywords:** *Sciatic nerve crush*, *Gallic acid*, *Exercise*, *Pain*, *Motor*, *SNCV*, *Rat*

## Abstract

**Objective:**

The aim of present study was to evaluate the effects of oral administration of gallic acid (GA) for 21 days alone and in combination with exercise on nerve conduction velocity and sensory and motor functions in rats with sciatic nerve crush.

**Materials and Methods:**

Seventy adult male Wistar rats (250-300 g) were divided randomly into 7 groups with 10 in each: 1) Control (Cont), 2) Crushed + Vehicle (Cr +Veh), 3-5) Crushed + gallic acid (Cr+GA) (50, 100, and 200 mg/kg/2 mL, orally), 6) Crushed + exercise (Cr+Exe), and 7) Crushed + exercise + effective dose of gallic acid (Cr+Exe +GA200) for 21 days. In order to establish an animal model of sciatic nerve crush, equivalent to 7 kg of force pressed on 2-3 mm of sciatic nerve for 30 s, three times with 30 s intervals. Pain sense reflex in hot plate, motor coordination in rotarod, and sciatic nerve conduction velocity (SNCV) in all groups were tested. Data were analyzed using one-way ANOVA followed by Tukey’s post hoc test and p<0.05 has assigned as the significant difference.

**Results:**

Pain threshold was increased significantly in untreated crushed rats while motor function and SNCV were decreased in all groups with nerve crush (p<0.05, p<0.01, p<0.001 vs. control). Pain reflex latency was not changed in treated groups. Motor coordination and SNCV were improved in groups Cr+GA200 and Cr+Exe + GA200 (p<0.05, p<0.01 vs. Cr+Veh).

**Conclusion:**

GA, dose-dependently, may have therapeutic potential to improve the peripheral nerve degeneration, which is most likely related, at least in part, to its antioxidant and therapeutic properties.

## Introduction

Peripheral nerves are often damaged by various circumstances including crush, compression, stretching, motor vehicle accidents, fractures, dislocations, and natural disasters such as earthquakes (Wong et al., 2011 ▶). After peripheral nerve injuries, the capability of injured axons to regenerate and recover functional connections is dependent on the age of the subject, the nerve trunk affected, the site and type of lesion, the type and delay of surgical repair and the distance over which axons must re-grow to span the injury. Thus, if a nerve transaction resulting in a gap between nerve stumps is left unrepaired or repaired with long grafts, the probability of effective re-innervations of muscle and sensory receptors are poor (Reyes et al., 2005 ▶). Within 6 hours of the injury, the nucleus migrates to the periphery of the cell and Nissl granules, rough endoplasmic reticulum break up and disperse. This process is called chromatolysis (Burnett and Zager, 2004 ▶). The transition between choromatolysis and apoptosis coincides with accumulation of metabolically active mitochondria and oxidative damage to nucleic acids and proteins in axotomized neurons. On the other hand, when their axons are effectively able to regenerate and reinnervate targets, neurons slowly return to a normal function and morphology (Navarro et al., 2007 ▶). Biochemical changes develop within hours after axotomy and coincide with the early chromatolytic morphological changes. The concentration of enzymes of the oxidative pentose phosphate shunt required for RNA synthesis is also raised (Harkonen and Kauffman, 1974 ▶), whereas production of neurotransmitters and cytoskeleton proteins is decreased (Hoffman and Lasek, 1980 ▶). The axotomized neurons shift from a transiting state to a regenerative state underlies by prominent changes in gene expression, which lead to a decrease in the synthesis of neurotransmission-related products and increased synthesis of growth-associated proteins and structural components of the membrane (Sharma et al., 2010 ▶). After peripheral nerve injury, the adult peripheral nervous system responds by increasing the availability of neurotrophic factors, either by autocrine or paracrine sources (Gambarotta et al., 2013 ▶). The spectrum of neurotrophic factors reported to stimulate axonal regeneration includes nerve growth factor (NGF), brain-derived neurotrophic factor (BDNF), neurotrophin-3 (NT-3) and neurotrophin -4/5 (NT-4/5), insulin-like growth factors (IGF-I and IGF-II), ciliary neurotrophic factor (CNTF), basic fibroblast growth factor (FGF-2), and glial cell line-derived neurotrophic factor (GDNF) (Navarro et al., 2007 ▶, Stassart et al., 2013 ▶).

## Materials and Methods

Seventy healthy adult male Wistar rats (250-300 g) obtained from Ahvaz Jundishapur University of Medical Sciences (AJUMS) Laboratory Animal Centre were used in this study. Animals were housed in standard cages under controlled room temperature (20±2 ˚C), humidity (55–60%), and light exposure conditions 12:12 h light–dark cycle (light on at 07:00 am). All experiments were carried out during the light phase of the cycle (8:00 am to 5:00 pm). Access to food and water was ad libitum except during the experiments. Animal handling and experimental procedures were performed under observance of the University and Institutional legislation, controlled by the Local Ethics Committee for the Purpose of Control and Supervision of Experiments on Laboratory Animals. All efforts were made to minimize animal suffering and reduce the number of animals used. Prior to the onset of behavioral testing, all rats were handled for 3 days (10 minutes daily). Animals were divided randomly into seven groups consisting of 10 animals in each group. 

1. Control: intact rats received normal saline (2 ml/kg) for 21 days.

2. Cr+Veh: sciatic nerve crushed rats received normal saline (2 ml/kg) for 21 days.

3. Cr+GA50: sciatic nerve crushed rats received 50 mg/kg/2 ml GA for 21 days.

4. Cr+GA100: sciatic nerve crushed rats received 100 mg/kg/2 ml GA for 21 days. 

5. Cr+GA200: sciatic nerve crushed rats received 200 mg/kg/2 ml GA for 21 days.

6. Cr+Exe: sciatic nerve crushed rats received forced exercise for 21 days.

7. Cr+Exe+GA200: sciatic nerve crushed rats received forced exercise combined with 200 mg/kg/2 ml GA as an effective dose for 21 days.

**Sciatic nerve crushing procedure**

We used the Kalender (2009) ▶ procedure for peripheral nerve crushing with little modification. Briefly, under general anesthesia with intraperitoneal ketamine (90 mg/kg) and xylazine (10 mg/kg), and after routine preparation of the operative field (hair trimming, 20% iodine ethylic alcohol solution), the right sciatic nerve was exposed through a 1-2 cm long posterolateral longitudinal straight incision on the lateral aspect of the right thigh followed by a gluteal muscle splitting incision. Sciatic nerve was exposed and with the use of needle holder equivalent to 7 kg of force pressed on 2-3 mm of sciatic nerve three times, 30 s with 30 s intervals. Penicillin procaine was applied to wound before closing the incision with sutures. All operations were performed on right limb (Bobinski et al., 2011 ▶). 

**Forced exercise training**

In order to physically train rats, we used the Sobral procedure (2008) with some modifications. The animals were given forced exercise (groups 6 and 7), 2 days after the surgery. Rats were put in a ten-channel treadmill once a day for 30 minutes for a period of 21 days with a constant inclination of zero. Rats were forced to run via a mild electric shock stimulation system installed on the end of its channels (3 mA). All animals ran at a speed of 17 m/min,) (Kalender et al., 2009 ▶, English et al., 2011 ▶). 

**Figure 1:**
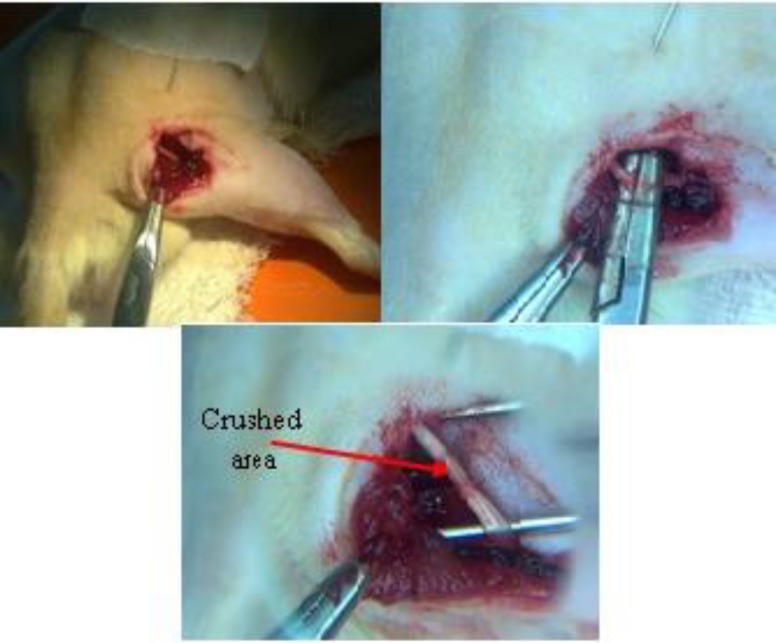
Serial photos to illustrate the surgery phases to induce the sciatic nerve crush in right leg of rat (left: expose the sciatic nerve, middle: crushing the nerve, right: showing the crushed area

**Figure 2:**
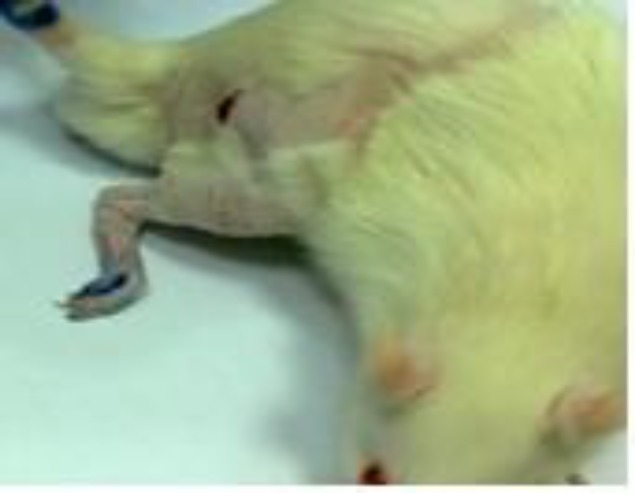
Photo to illustrate foot and fingers expand states in crushed sciatic nerve

**Treatment**

Treated rats received different doses of GA dissolved in 2 ml normal saline per 1000 g body weight orally (i.g.) every day 8:00–9:00 am for 21 days, starting from 2^nd^ day after nerve crush surgery. Sham-treated animals (Cr+Veh) received the same volume of normal saline for the same period.

**Sciatic nerve conduction velocity (SNCV)**

At the 21^st^ day after nerve crush injury, the SNCV was performed under general anesthesia, and the sciatic nerve was stimulated with stimulus intensity through bipolar needle electrodes, proximal to the injury site at the level of the sciatic notch, and recording was done from two points distal to the lesion at the level of the ankle and knee. Square wave stimulus pulses of 500 μs in duration were delivered at 1 Hz. 

The latency of the evoked muscle twitch was recorded from the intrinsic foot muscles with bipolar needle electrode (Nihon Kohden Co., Japan) amplified and recorded by 4-channels PowerLab system (AD Instrument Co., Australia). Finally, the distance between the two points of stimulating electrodes on the skin with a ruler and latencies of action potentials were measured. Conduction velocity was calculated using the following formula. 

SNCV=Δd/ Δt

which the SNCV is motor nerve conduction velocity (cm/ms), Δd is the difference between distances of two stimulatory electrodes at points of ankle and knee respectively, and Δt is the difference between delay times of recorded compound action potentials at points of ankle and knee respectively (Kalender et al., 2009 ▶, Teodori et al., 2011 ▶, Udina et al., 2011 ▶).

**Statistical analysis**

Data were expressed as mean±SEM. Statistical analysis was performed using one-way analysis of variance (ANOVA) followed by Tukey’s post hoc test. *P *values less than 0.05 were assumed to denote a significant difference.

**Figure 3:**
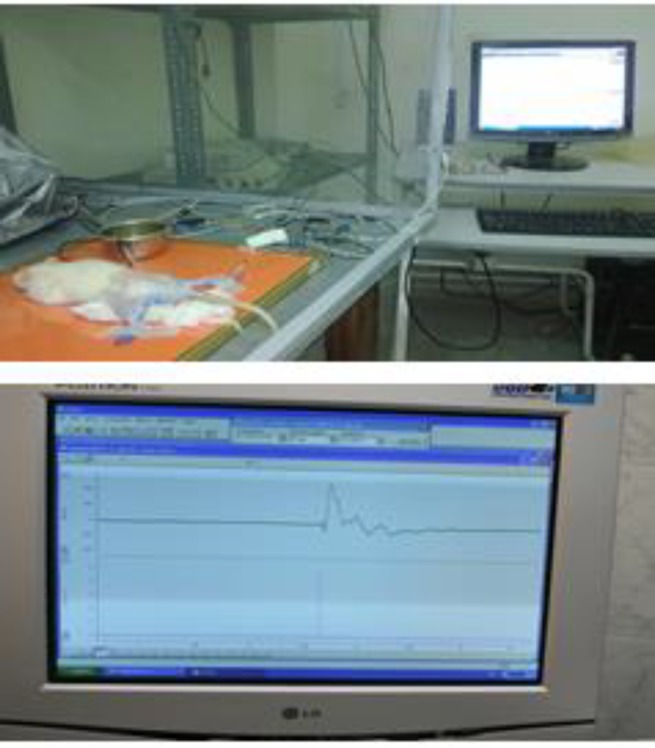
Top: SNCV recording set up using PowerLab recorder. Down: a typical recorded compound action potential from distal innervated muscles after stimulating the proximal part of sciatic nerve

## Results

**Pain sense reflex latency**

**Figure 4:**
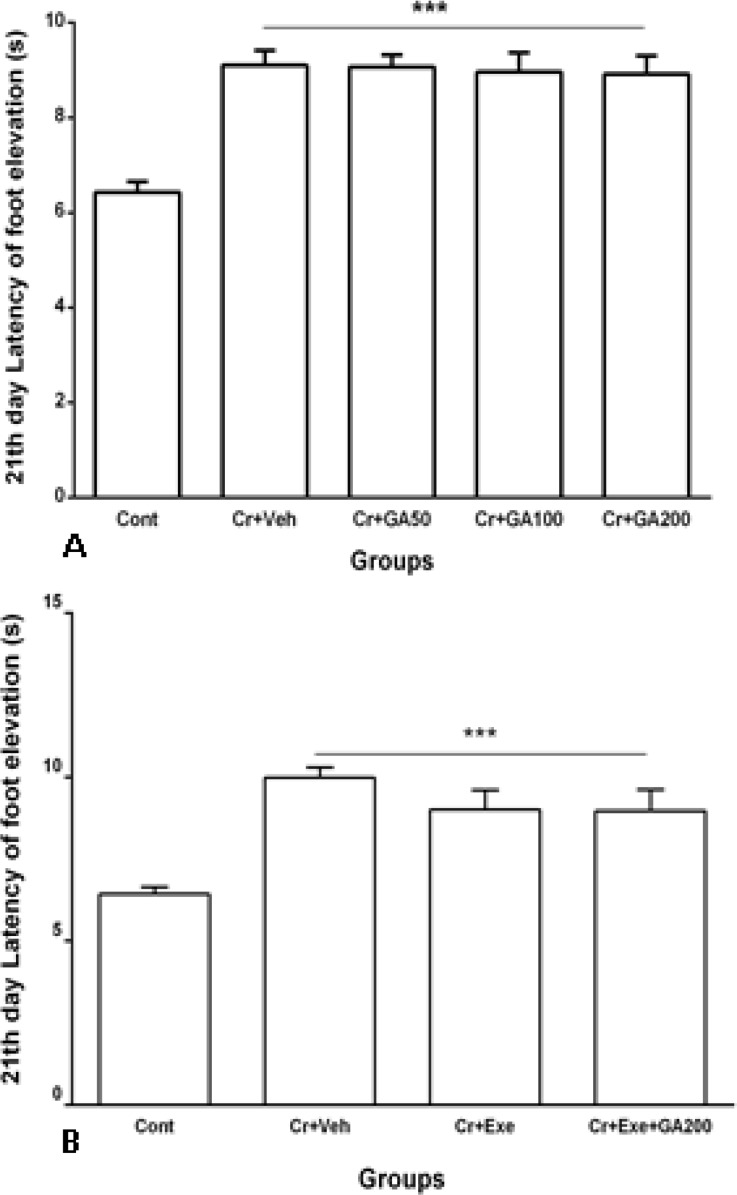
Latency of lifting foot in hot plate (as pain assessment) at 21^st^ day after sciatic nerve crush in Control, Cr+Veh, Cr+GA50, Cr+GA100, Cr+GA200 (A), Cr+Exe, and Cr+Exe+GA200 groups (B). Values are expressed as mean±SEM and analyzed using one-way ANOVA followed by Tukey's post hoc test (n=10). *** *p*<0.001for treated groups vs. control

As shown in Figure 4, delay of lifting foot increased significantly (p<0.001) in rats with crushed sciatic nerve 21 days after treatment with vehicle or different doses of gallic acid even with stepping on heated plate for longer time when compared with control group. Treatment with GA could not improve the sciatic nerve crush-induced analgesia (Figure 4A). Physical exercise alone and also combined with most effective dose of GA for 21 days (Cr+Exe and Cr+Exe+GA200 groups) had no effect on impaired pain sense after crushing (Figure 4B). Therefore, treatment with GA or physical exercise could not improve the sciatic nerve crush-induced analgesia. 

**Motor coordination**

**Figure 5:**
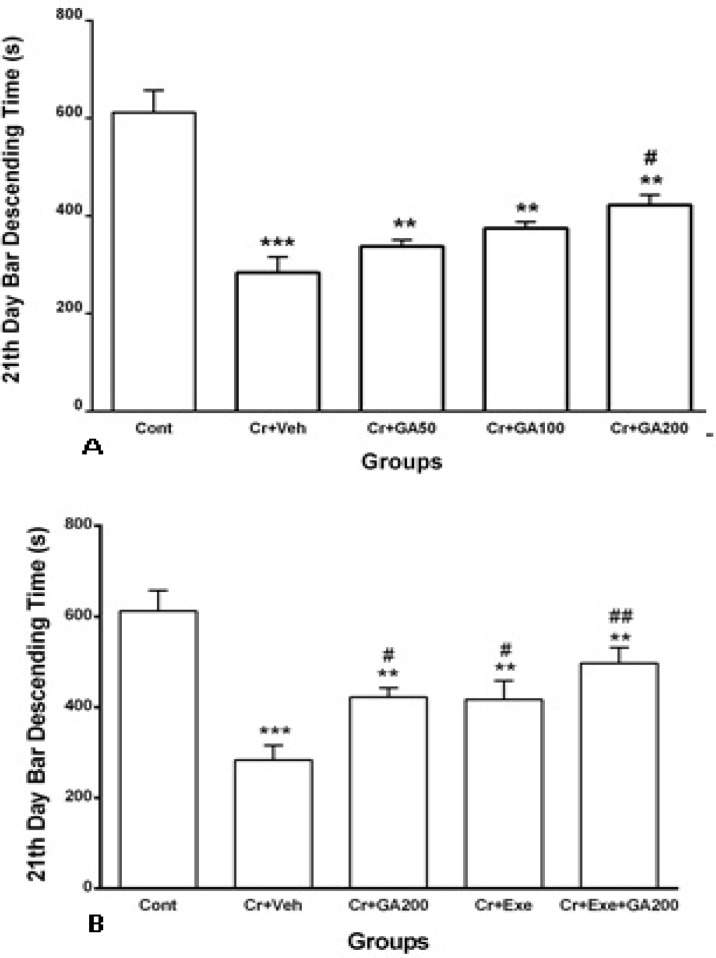
Mean±SEM of duration of falling on a rotating rod in rotarod (bar descending as motor coordination assessment) at 21^st^ day after sciatic nerve crush in Control, Cr+Veh, Cr+GA50, Cr+GA100, Cr+GA200, Cr+Exe, and Cr+Exe+GA200. Data were analyzed using one-wayANOVA followed by Tukey's post hoc test (n=9). ***p<0.001 and **p<0.01 for crushed groups vs. Control, #p<0.05 for Cr+GA200 and ##p<0.01 for Cr+Exe+GA200 vs. Cr+Veh, respectively

As shown in Figure 5, the latency of falling on a rotating rod decreased significantly (p<0.001, p<0.01) in crushed sciatic nerve groups 21 days after treatment with vehicle or different doses of gallic acid when compared with control group. Treatment with different doses GA increased latency of falling on a rotating rod but only dose 200 mg/kg GA caused significant increase in this latency (p<0.05, Figure 5A). Physical exercise alone and also combined with most effective dose of GA for 21 days (Cr+Exe and Cr+Exe+GA200 groups) could improve significantly latency of falling on a rotating rod (p<0.05, p<0.01, Figure 5B), but their latency was still lower than control rats.

**Sciatic nerve conduction velocity (SNCV)**

**Figure 6:**
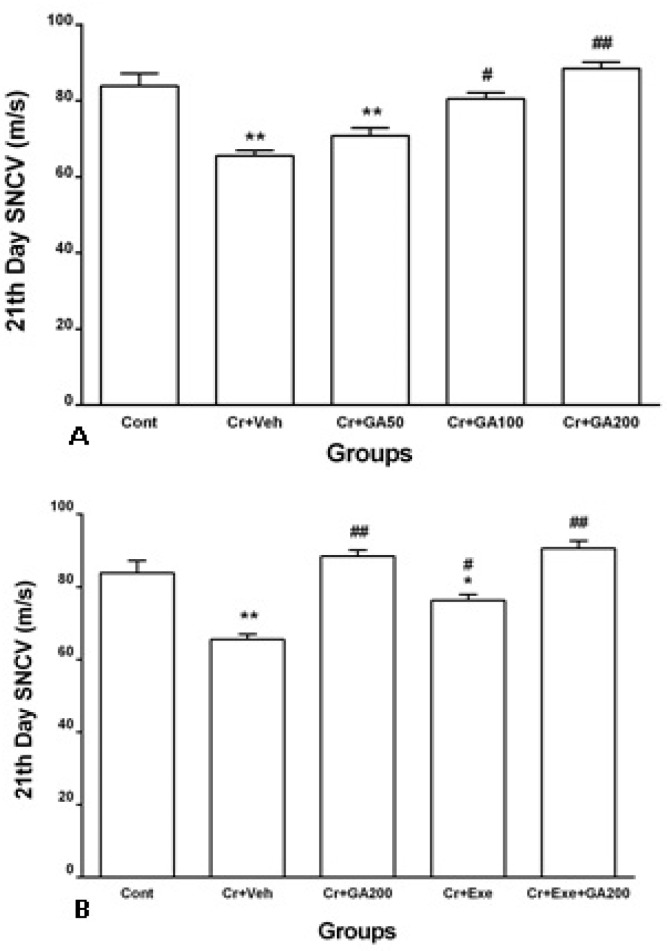
Effect of different doses of gallic acid and exercise alone and most effective dose of GA combined with exercise for 21 days on sciatic nerve conduction velocity (SNCV) in different groups including Control, Cr+Veh, Cr+GA50, Cr+GA100, Cr+GA200, Cr+Exe, and Cr+Exe+GA200. Values are expressed as mean±SEM. Data were analyzed using one way ANOVA followed by Tukey’s post hoc test (n=9). * P< 0.05, ** p< 0.01 vs. Control and #p<0.05, ##p<0.01 vs. Cr+Veh

As shown in Figure 6, the SNCV decreased significantly (p<0.01, p<0.05) in rats with crushed sciatic nerve received normal saline (vehicle of GA) or physical exercise alone for 21 days after nerve crushing. Treatment of crushed rats with low dose of GA did not change SNCV while doses of 100 and 200 mg/kg alone could increase it significantly (p<0.05, p<0.01, Figure 6A). Physical exercise alone and also combined with most effective dose of GA for 21 days (Cr+Exe and Cr+Exe+GA200 groups) could improve SNCV significantly (p<0.05, p<0.01, Figure 6B), Improved SNCV in Cr+GA200 and Cr+Exe+GA200 groups was similar in control rats.

## Discussion

The results of current work showed that pain sense reflex latency after sciatic nerve crush was increased significantly and treatment with GA alone and combine with exercise could not reverse it. Motor coordination was impaired after sciatic nerve crush significantly, while treating with dose of 200 mg/kg GA could improve it at 21^st^ day after sciatic nerve crush. Treated crushed animals with dose of 200 mg/kg GA alone and combined with exercise improved significantly the motor coordination at 21^st^ day after sciatic nerve crush. 

Sciatic nerve conduction velocity (SNCV) was impaired significantly in rats with sciatic nerve crush treated with normal saline (vehicle) for 21 days. Treated crushed animals with doses of 100 and 200 mg/kg GA or physical exercise alone and also effective dose GA combined with exercise improved it significantly. 

 Lesions of peripheral nerves cause loss of motor and sensory function and also lead to hyper reflexes and hyperalgesia (Teodori et al., 2011 ▶; Udina et al., 2011 ▶). After injuries to peripheral nerves, axons and myelin sheaths distal to the lesion are degraded. The degenerative products are eliminated by the cooperative action of denervated Schwann cells and infiltrating macrophages. Valerian degeneration serves to create a microenvironment favoring axonal regrowth. Schwann cells within the endoneurial tubes of the distal nerve dedifferentiate towards a non-myelinating proliferative phenotype that over-express growth factors, cell adhesion molecules, and extracellular matrix to promote regeneration (Mohammadi et al., 2013 ▶). The sciatic nerve of rats is a reliable model for studying different kinds of injuries and treatment methods, with crushing injuries being one of the preferred types, because it causes rupture of nervous fibers without rupturing most of the nerve’s supporting structures, enabling an easier regeneration after injury (Monte-Raso et al., 2009 ▶). Although crushed peripheral nerves keep anatomical continuity, regenerate spontaneously, and somehow reinnervate their target tissues, the longer it takes for the crushed nerve to reinnervate their target tissues, the greater the chance of permanent denervation atrophy of the target tissues. In this work our results showed that the latency of pain reflex was too long. Our finding is inconsistent with Rodriguez et.al, (2004) and Monte-Raso et.al, (2009) ▶. Difference between our findings with other investigators may be due to using different types and severity of crushing. 

Therefore, accelerated nerve regeneration is crucial to obtain satisfactory functional outcomes (Yuan et al., 2010 ▶). Traditionally, functional nerve defects have been remedied by many methods, including nerve transfer, nerve grafts, artificial nerve conduit bridging, and end-to-side neurorrhaphy (Oberlin et al., 1994 ▶). However, these methods only provide a regenerative environment for injured nerves. Recovery of function depends on various local and systemic factors. Regeneration of axons from the proximal stump of an injured nerve to the distal nerve stump is one of the most important factors in reinnervation of peripheral tissue. 

Recent studies have shown that locally applied neurotrophins can enhance survival of damaged neurons and regrowth of lesioned axons in the central and peripheral nervous systems in rats. Activity-dependent therapies promote axonal regeneration and functional recovery and may improve sensory–motor coordination and restoration of adequate circuitry at the spinal level. 

Maintenance of denervated muscle activity and afferent input, by active or passive exercise, may increase trophic factor release to act on regenerating axons and to modulate central neuronal plasticity (Chen et al., 2001 ▶; English et al., 2011 ▶). Its effects are related to the increased expression of brain-derived neurotrophic factor (BDNF) and its receptor, tyrosine kinase B (trkB) (Gomez-Pinilla et al., 2001 ▶). BDNF, glial cell line-derived neurotrophic factor (GDNF), neurotrophin-3 (NT-3), and neurotrophin-4 (NT-4) act as chemo-attractants for regenerating axons (Nothias et al., 2005 ▶) and are capable of modifying neuronal circuits by strengthening and increasing the formation of synapses (Gomez-Pinilla et al., 2002 ▶). 

In vivo studies have shown that local continuous release of BDNF increases nerve fiber growth over time and induces remyelination and faster nerve regeneration in rats after sciatic nerve injury. BDNF enhances the expression of myelin-associated glycoprotein (MAG) and glycoprotein zero (P0) (Vogelin et al., 2006 ▶). Our results showed that treatment of rats with sciatic nerve crush with higher dose of GA or chronic exercise alone or combination of GA with exercise improved the motor coordination and SNCV significantly. 

Physical activity alters the structure and function of the neuromuscular junction (NMJ). However, exercise increases the size and the degree of branching of motor nerve terminals at the NMJ, increases the total area of both pre- and postsynaptic elements, and increases the quantal content of acetylcholine release (Bobinski et al., 2011 ▶). 

Increased amount of mRNA expression of caspase in spinal dorsal root ganglion (DRG) neuron following SCI was reduced by exercise (Keeler et al., 2012 ▶). Exercise, whether in the acute or late phase of injury, also positively influenced the maturation of regenerated nerve fibers. After axonotmesis, axon diameters can reach control values after six months (Schröder, 1972 ▶). Teodori et al., 2011, reported that one month after injury, the nerve fiber diameter of exercised sciatic nerve injury rats reached 68-70% of control values (Teodori et al., 2011 ▶). 

It is known that after the injury due to the tissue destruction, free oxygen radicals increase and cause tissue damage (Bagdatoglu et al., 2002 ▶). Neuropathic pain was developed by impairing the normal functions of myelinated and unmyelinated fibers in sciatic nerve. Gallic acid is one of phenolic acids that is found in green tea and grape seeds (Kim et al., 2006 ▶). Its antioxidant activity is closely associated with activity against various cancer types, cardiovascular diseases, several dermal disorders cytoprotective, and anti-inflammatory action (Kaur et al., 2009 ▶). 

Antioxidant supplementation may provide neuroprotection against age-related neurodegenerative disorders, including Parkinson’s disease, amyotrophic lateral sclerosis, and Alzhimer’s disease (Tavares et al., 2013 ▶). Flavonoids exert a multiplicity of neuroprotective actions within the brain, including a potential to protect neurons against injury induced by neurotoxins, an ability to suppress neuroinflammation, and the potential to promote memory, learning, and cognitive function. These effects appear to be underpinned by two common processes. Firstly, they interact with critical protein and lipid kinase signalling cascades in the brain leading to an inhibition of apoptosis triggered by neurotoxic species and to a promotion of neuronal survival and synaptic plasticity. Secondly, they induce beneficial effects on the vascular system leading to changes in cerebrovascular blood flow capable of causing angiogenesis, neurogenesis, and changes in neuronal morphology (Vauzour et al., 2008 ▶). 

*Punica granatum *rind is composed of phenolic punicalagins, gallic acid, catechin (Amakura et al., 2000 ▶), and various flavanoids such as quercetin and rutin (Gómez-Caravaca et al., 2013 ▶) which the latter has been documented to possess free radical scavenging property (Rosenblat et al., 2006 ▶). Treatment with aqueous extract of *P. granatum *(100 mg/kg, p.o.) markedly prevented sciatic nerve ligation-induced neuropathy and oxidative stress by changing the pain threshold, improving the functionality of sciatic nerve, decreasing serum and tissue thiobarbituric acid reactive substances (TBARS) and tissue superoxide dismutase (SOD), and increasing levels of serum glutathione and catalase. It may be concluded that *P. granatum *extract reduced the oxidative stress via inhibiting mitogen-activated protein kinase (MAPK), alleviated neuropathic symptoms, and consequently improved the functionality of sciatic nerve and prevented sciatic nerve ligation–induced neuropathic pain (Sukhvir Kaur et al., 2012 ▶).

Our data showed that oral administration of GA alone and combined with exercise for 21 days could not improve the pain sense reflex after sciatic nerve injury, while improved the impaired motor coordination and SNCV significantly in rats with sciatic nerve injury. Further investigations need to determine the exact mechanism(s) of the effect of GA and physical activity on peripheral nerve crush. 
